# Associations of plasma soluble CD22 levels with brain amyloid burden and cognitive decline in Alzheimer’s disease

**DOI:** 10.1126/sciadv.abm5667

**Published:** 2022-04-01

**Authors:** Xian-Le Bu, Pu-Yang Sun, Dong-Yu Fan, Jun Wang, Hao-Lun Sun, Yuan Cheng, Gui-Hua Zeng, Dong-Wan Chen, Hui-Yun Li, Xu Yi, Ying-Ying Shen, Luke A. Miles, Paul Maruff, Ben J. Gu, Christopher J. Fowler, Colin L. Masters, Yan-Jiang Wang

**Affiliations:** 1 Department of Neurology and Centre for Clinical Neuroscience, Daping Hospital, Third Military Medical University, Chongqing, China.; 2 Institute of Brain and Intelligence, Third Military Medical University, Chongqing, China.; 3 Chongqing Key Laboratory of Ageing and Brain Diseases, Chongqing, China.; 4 Shigatse Branch, Xinqiao Hospital, Third Military Medical University, Shigatse, China.; 5 The Florey Institute, The University of Melbourne, Parkville, Victoria, Australia.; 6 CogState, Melbourne, Victoria, Australia.; 7 State Key Laboratory of Trauma, Burns and Combined Injury, Third Military Medical University, Chongqing, China.; 8 Center for Excellence in Brain Science and Intelligence Technology, Chinese Academy of Sciences, Shanghai, China.

## Abstract

CD22 has been suggested to contribute to Alzheimer’s disease (AD) pathogenesis by inhibiting microglial amyloid β (Aβ) phagocytosis. Soluble CD22 (sCD22) generated by cleavage from cell membranes may be a marker of inflammation and microglial dysfunction; but alterations of sCD22 levels in AD and their correlation with AD biomarkers remain unclear. Plasma sCD22 levels were measured in cognitively normal non-AD participants and patients with preclinical AD and AD dementia from a Chinese cohort and the Australian Imaging, Biomarkers and Lifestyle Flagship Study of Ageing. Plasma sCD22 levels were elevated in patients with preclinical and dementia AD. Plasma sCD22 levels were negatively correlated with cerebrospinal fluid (CSF) Aβ42 levels and Aβ42/Aβ40, and positively correlated with CSF phosphorylated tau levels and brain Aβ burden, but negatively correlated with cognitive function. Moreover, higher plasma sCD22 levels were associated with faster cognitive decline during follow-up. These findings suggest that CD22 plays important roles in AD development, and that sCD22 is a potential biomarker for AD.

## Teaser

Plasma soluble CD22 levels were increased in Alzheimer’s disease and associated with brain amyloid burden and cognitive decline.

## INTRODUCTION

Alzheimer’s disease (AD) is the most common form of dementia. The number of patients with AD continues to increase with the aging of the population causing a heavy burden on families and societies (*1*). The senile plaques formed by the amyloid β (Aβ) peptide and neurofibrillary tangles consisting of hyperphosphorylated tau proteins in the brain are the pathological hallmarks of AD (*2*, *3*). Microglia mediate crucial functions to support the central nervous system and they play key roles in the pathogenesis of AD. It is known that microglia are able to clear protein aggregates and cellular debris to maintain cerebral homeostasis (*4*). This function is impaired in AD, with studies showing that the reduced Aβ phagocytic capacity of microglia may be a mechanism underlying AD development (*5*). However, the mechanisms of impaired microglial homeostatic function in AD remain unclear. A wide range of receptors expressed on microglia recognize exogenous or endogenous stimuli and initiate innate immune responses (*5*). Genome-wide association studies on large sporadic AD populations have found that rare coding variants in PLCG2, ABI3, TREM2, and CD33 expressed on microglia are associated with a higher risk of AD (*5*–*7*).

Sialic acid–binding immunoglobulin-like lectin (Siglec) receptors on microglia can inhibit phagocytosis when activated by sialic acid–bearing entities (*7*, *8*). CD22, also called siglec-2, was previously thought to be mainly expressed on mature B cells (*9*). It was found that CD22 is a negative regulator of microglial phagocytosis in the brain, and that inhibition of CD22 promotes microglial phagocytosis of Aβ oligomers and lessens cognitive impairment in aged mice (*10*). A recent study revealed that CD22 was expressed in oligodendrocytes in the human brain and that its receptor was expressed on microglia (*11*). Soluble CD22 (sCD22) is generated by the cleavage of the extracellular domain of CD22. Several studies indicate that sCD22 may be a marker of inflammation and microglial dysfunction (*11*–*13*). Thus, questions arise as to whether CD22 is involved in the pathogenesis of AD and whether the level of sCD22 changes pathologically in AD. In the present study, we aimed to test the alteration of sCD22 levels in blood from cognitively normal non-AD (CN) participants and patients who had either preclinical or clinical AD and investigate the association of sCD22 with brain Aβ deposition and cognitive decline in two independent cohorts.

## RESULTS

### Characteristics of the participants

The demographic and clinical features of the Chongqing cohort are shown in table S1. There was no difference in age or education levels among the CN, preclinical AD, and AD dementia groups. The AD dementia group had a higher percentage of female participants and apolipoprotein E (APOE) ε4 carriers. The median Mini-Mental State Examination (MMSE) score for the AD dementia group was lower than that of CN and preclinical AD participants. As expected, patients with preclinical AD had lower cerebrospinal fluid (CSF) Aβ42 and Aβ40 levels and higher total tau (t-tau) and phosphorylated tau (p-tau) 181 levels than CN participants.

In the Australian Imaging, Biomarkers and Lifestyle (AIBL) cohort (table S2), preclinical AD and AD dementia participants were older and had a higher frequency of APOE ε4 carriers than CN participants, but there were no differences among the three groups in sex or education level. The median MMSE score of the AD dementia group was lower than those of the CN and preclinical AD groups. The episodic memory (EM) and Preclinical Alzheimer Cognitive Composite (PACC) scores of the preclinical AD and AD dementia groups were both lower than those of the CN group.

### Plasma sCD22 levels in the Chongqing and ABIL cohorts

**Fig. 1.**
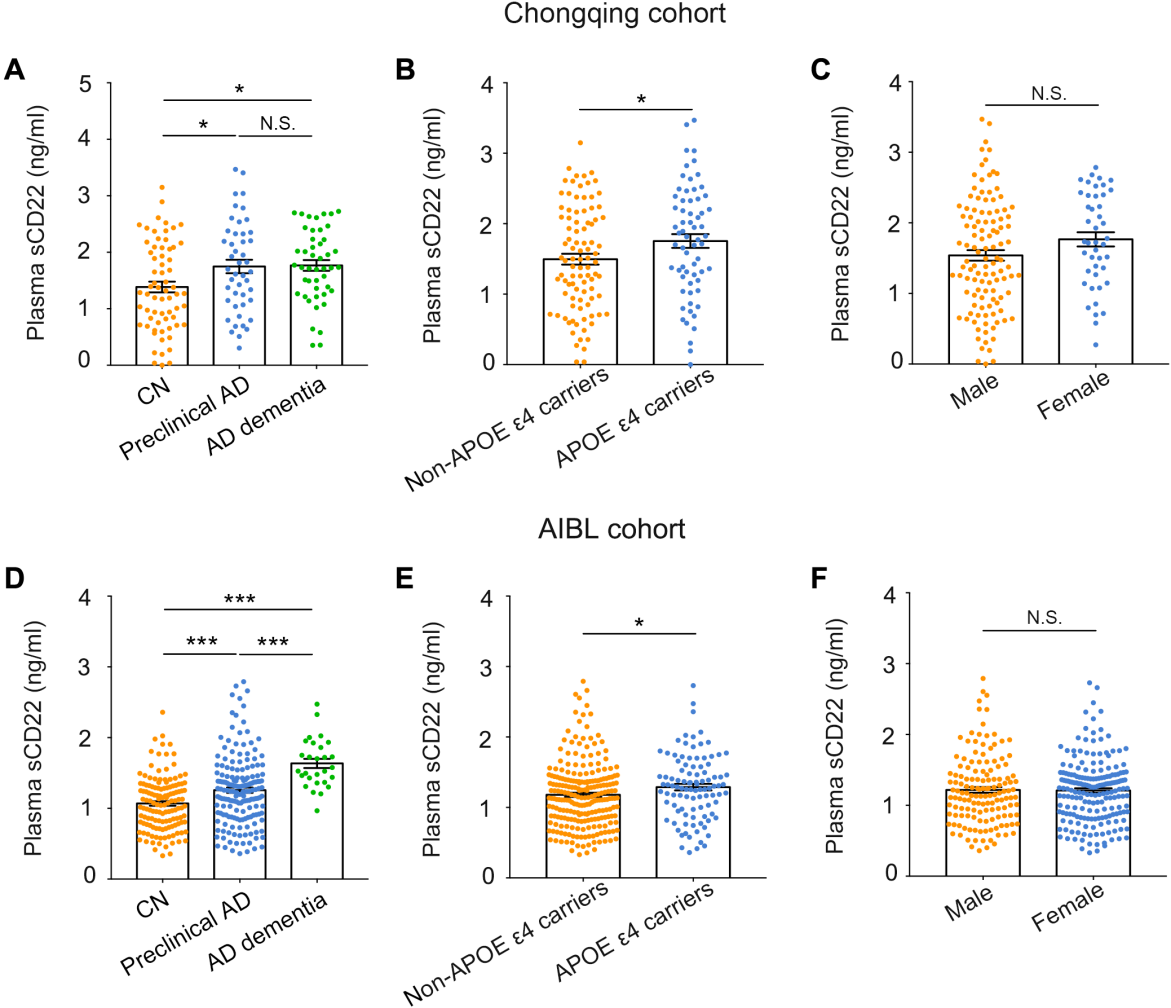
Plasma levels of sCD22 in the Chongqing and AIBL cohorts. Comparison of plasma sCD22 levels between cognitively normal non-AD (CN), preclinical AD, and AD dementia participants in the Chongqing cohort (**A**) and AIBL cohort (**D**). Comparison of plasma sCD22 levels between non-APOE ε4 carriers and APOE ε4 carriers in the Chongqing cohort (**B**) and AIBL cohort (**E**). Comparison of plasma sCD22 levels between males and females in the Chongqing cohort (**C**) and AIBL cohort (**F**). *P* values are indicated with asterisks: *P < 0.05 and ***P < 0.001. N.S. denotes no significant difference.

We tested the plasma levels of sCD22 in both the Chongqing and AIBL cohorts. In the Chongqing cohort, the plasma sCD22 levels in patients with preclinical AD (1748.7 ± 808.3 pg/ml versus 1385.1 ± 775.3, *P* < 0.05) and AD dementia (1766.7 ± 639.7 pg/ml versus 1385.1 ± 775.3, *P* < 0.05) were both higher than those in CN participants (Fig. 1A). In the AIBL cohort, patients with preclinical AD (1257.6 ± 491.1 pg/ml versus 1069.2 ± 376.2, *P* < 0.001) and patients with AD dementia (1635.6 ± 343.3 pg/ml versus 1069.2 ± 376.2, *P* < 0.001) also had higher plasma sCD22 levels than CN participants, with plasma sCD22 levels in patients with AD dementia higher than those in patients with preclinical AD (1635.6 ± 343.3 pg/ml versus 1257.6 ± 491.1, *P* < 0.001) (Fig. 1D). We also compared plasma sCD22 levels by analysis of covariance (ANCOVA) with adjustment for age, sex, APOE ε4 genotype, and comorbidities, and the results remained essentially the same (table S3).

We further investigated whether plasma sCD22 levels were related to age, sex, and APOE ε4 genotype. No correlation of plasma sCD22 with age was observed in the Chongqing cohort or AIBL cohort (fig. S1). Plasma sCD22 concentrations were elevated in APOE ε4 carriers compared to non-APOE ε4 carriers (Fig. 1, B and E) but were not affected by sex in either the Chongqing or AIBL cohorts (Fig. 1, C and F).

### Correlations of plasma sCD22 with AD core biomarkers in the Chongqing cohort

**Fig. 2.**
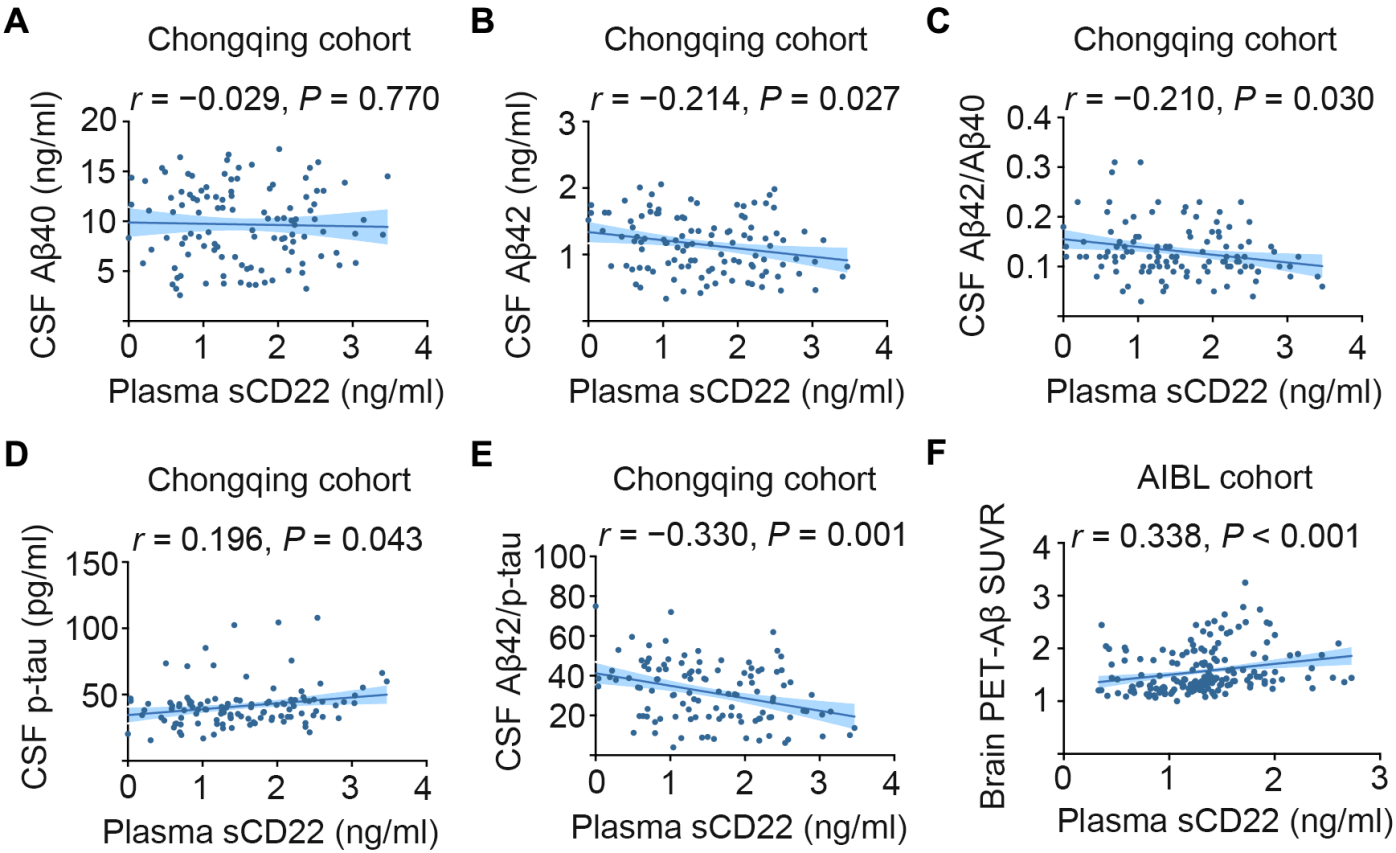
Correlation of plasma sCD22 with AD core biomarkers and brain Aβ burden. (**A** to **E**) Scatter plots representing the associations of plasma sCD22 with each of the AD CSF biomarkers in the Chongqing cohort. (**F**) Scatter plots representing the associations of plasma sCD22 with brain Aβ burden reflected by Aβ–positron emission tomography (PET) standard uptake value ratio (SUVR) in the AIBL cohort. Partial correlation analyses are adjusting for age, sex, APOE ε4 genotype, and comorbidities. The shaded areas represent the 95% confidence intervals.

The associations between sCD22 and AD core markers in the Chongqing cohort were analyzed by partial correlation analyses with adjustment for age, sex, APOE ε4 genotype, and comorbidities. The plasma sCD22 level was negatively correlated with the CSF Aβ42 level (*r* = −0.214, *P* = 0.027) and the ratio of CSF Aβ42 to Aβ40 (*r* = −0.210, *P* = 0.030) as well as CSF Aβ42 to p-tau (*r* = −0.330, *P* = 0.001) but positively correlated with the CSF p-tau level (*r* = 0.196, *P* = 0.043) (Fig. 2, A to E). No correlations of plasma sCD22 with CSF t-tau or the ratio of CSF p-tau to t-tau were found (fig. S2).

### Correlation of plasma sCD22 levels with Aβ burden in the brain

We examined the association between plasma sCD22 levels and brain Aβ burden as a continuous variable. As shown in Fig. 2F, plasma sCD22 levels were positively correlated with brain Aβ burden as reflected by ^11^C Pittsburgh Compound B–positron emission tomography (PiB-PET) standard uptake value ratio (SUVR) (*r* = 0.338, *P* < 0.001) in the AIBL cohort after adjustment for age, sex, and APOE ε4 genotype.

### Associations between baseline plasma sCD22 levels and cognitive function

**Fig. 3.**
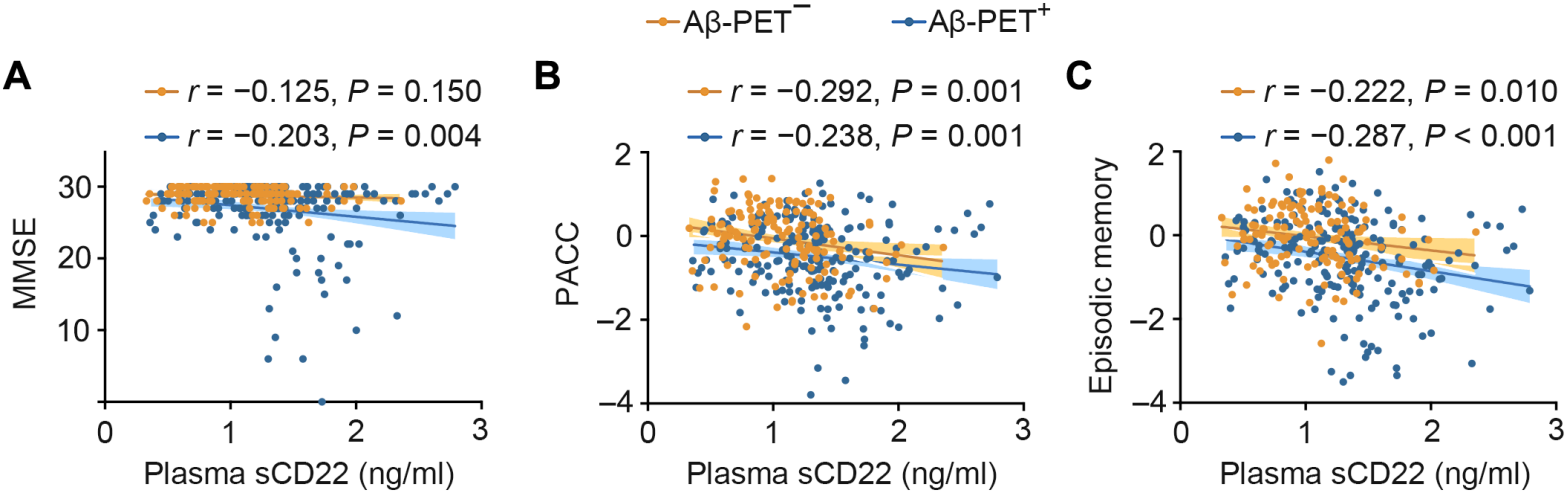
Associations between baseline plasma sCD22 levels and cognitive function in the AIBL cohort. Scatter plots representing the correlations of plasma sCD22 with MMSE (**A**), PACC (**B**), and EM (**C**) scores in the Aβ-PET–negative and Aβ-PET–positive groups. Partial correlation analyses are adjusting for age, sex, and APOE ε4 genotype. The shaded areas represent the 95% confidence intervals.

**Fig. 4.**
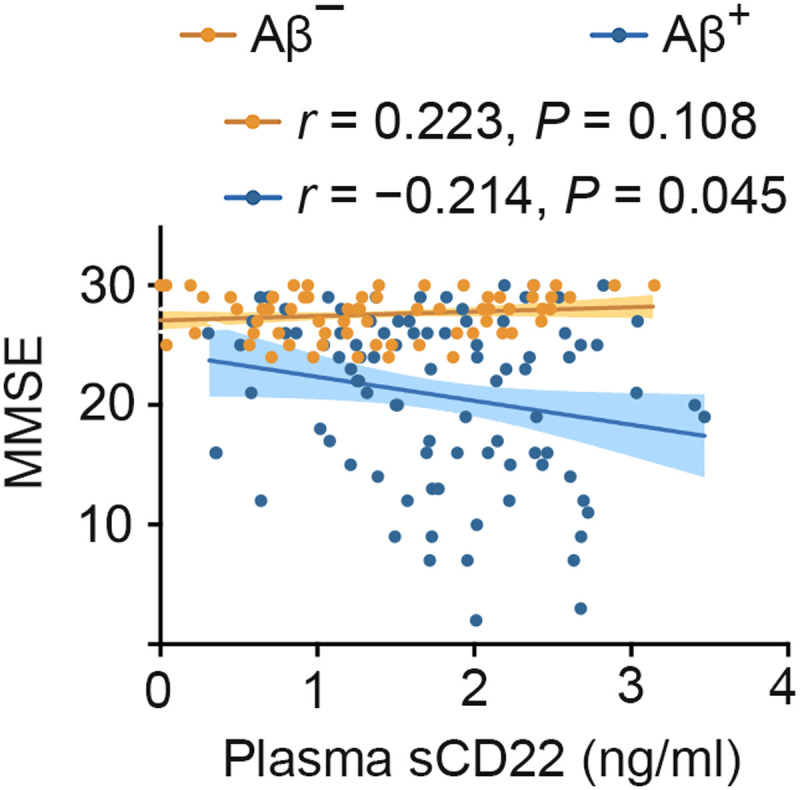
Associations of plasma sCD22 levels with baseline MMSE scores in the Chongqing cohort. Participants in the Chongqing cohort were binarized into brain Aβ-negative (A^−^) or Aβ-positive (A^+^) groups on the basis of their CSF Aβ42 levels or Aβ-PET imaging. Participants with normal measures of CSF Aβ42 or negative Aβ-PET were classified as A^−^. Participants with positive CSF Aβ42 or Aβ-PET were classified as A^+^. Partial correlation analyses are adjusting for age, sex, APOE ε4 genotype, and comorbidities. The shaded areas represent the 95% confidence intervals.

In the AIBL cohort, higher plasma sCD22 levels were correlated with lower MMSE (*r* = −0.203, *P* = 0.004), PACC (*r* = −0.238, *P* = 0.001), and EM (*r* = −0.287, *P* < 0.001) scores in the Aβ-PET^+^ group and were correlated with lower PACC (*r* = −0.292, *P* = 0.001) and EM (*r* = −0.222, *P* = 0.010) scores in the Aβ-PET^−^ group after adjustment for age, sex, and APOE ε4 genotype (Fig. 3). In the Chongqing cohort, higher plasma sCD22 levels were also correlated with lower MMSE scores (*r* = −0.214, *P* = 0.045) in the Aβ-positive group but not in the Aβ-negative group (Fig. 4). These results suggest that higher plasma sCD22 levels are associated with worse cognition at baseline.

### Associations between baseline plasma sCD22 levels and the subsequent cognitive decline rates

**Fig. 5.**
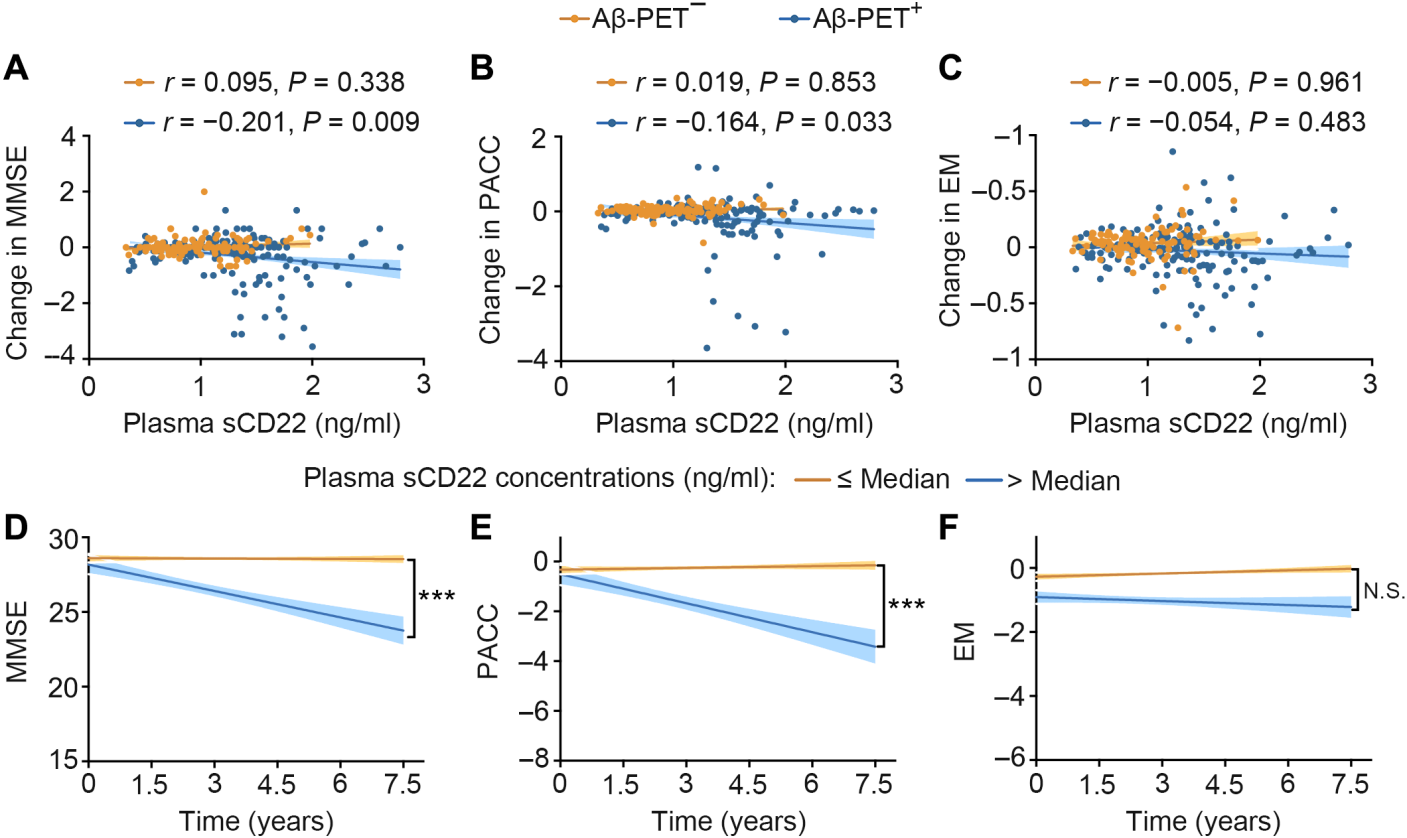
Associations between plasma sCD22 levels and the rate of cognitive decline in the AIBL cohort. (**A** to **C**) Scatter plots representing the correlations of plasma sCD22 levels with the decline of MMSE, PACC, and EM in the Aβ-PET–negative and Aβ-PET–positive groups. Partial correlation analyses are adjusting for age, sex, and APOE ε4 genotype. The shaded areas represent the 95% confidence intervals. (**D** to **F**) Plasma sCD22 levels (low versus high) and time interaction were assessed in the Aβ-PET–positive group only. The top (yellow) line is for low levels of sCD22, and the bottom (blue) line is for high levels of sCD22. Linear mixed model with adjustment for age, sex, and APOE ε4 genotype. ***P < 0.001.

We also investigated the associations between baseline plasma sCD22 levels and the rates of subsequent cognitive decline in the AIBL cohort. As shown in Fig. 5, A to C, baseline plasma sCD22 levels were correlated with the subsequent change in MMSE (*r* = −0.201, *P* = 0.009) and PACC (*r* = −0.164, *P* = 0.033) scores in the Aβ-PET^+^ group but not in the Aβ-PET^−^ group after adjustment for age, sex, and APOE ε4 genotype. We next tested whether higher baseline plasma sCD22 levels predicted longitudinal cognitive decline in the Aβ-PET^+^ group. To this end, we computed a linear mixed model (adjusted for age, sex, and APOE ε4 genotype) to test the interaction between plasma sCD22 levels and time on cognitive scores. As shown in Fig. 5, D to F, higher baseline plasma sCD22 levels were associated with a faster decline in MMSE (*P* < 0.001) and PACC (*P* < 0.001) scores over time in the Aβ-PET^+^ group.

## DISCUSSION

Our study found that plasma sCD22 was elevated in patients with AD, and that its level was correlated with brain Aβ burden, CSF p-tau levels, and baseline cognitive impairment. Plasma sCD22 levels could predict the longitudinal cognitive decline over a 7.5-year follow-up. These findings suggest that sCD22 may play an important role in the pathogenesis of AD.

Microglia play crucial roles in maintaining brain homeostasis, such as clearing cellular debris and regulating synapse formation (*14*). Microglial dysfunction is a major component of AD pathogenesis (*15*, *16*). The failure to clear Aβ has been thought to be the main reason for brain Aβ accumulation in sporadic AD (*17*). Microglia play a central role in the clearance of Aβ in the brain. In the AD brain, the capacity of microglia to clear Aβ is largely compromised (*18*). However, the underlying mechanisms remain unclear. Recent studies have reported that CD22 is a negative regulator of microglial phagocytosis of Aβ, and sCD22 impairs the lysosome function of microglia (*10*, *11*). In our study, we found that plasma sCD22 was elevated in patients with AD and was associated with brain Aβ deposition and cognitive decline, implying that increased CD22 expression is a potential reason for the dysfunction of microglia in the AD brain. As sCD22 levels were associated with longitudinal cognitive decline in our study, it is intriguing to speculate that increased CD22 may also be involved in microglia-mediated synapse loss in AD. Our findings imply that CD22 might play an important role in the dysfunction of microglia in AD. This needs to be investigated in the future.

In addition, CD22 expression in microglia was up-regulated during aging and in AD (*10*, *19*, *20*). Several studies have shown that inhibition of CD22 promotes Aβ clearance by microglia and improves cognitive function (*10*, *20*). A recent study found that blocking the interaction between sCD22 and microglia improved lysosome function in human pluripotent stem cell–derived microglia-like cells with Niemann-Pick type C patient mutation (*11*). These findings suggest that sCD22 may represent a potential therapeutic target for AD.

The AT(N) system has been proposed for use as biomarkers to chart core AD pathophysiological features, namely, the Aβ pathway (A), tau-mediated pathophysiology (T), and neurodegeneration (N). As neuroinflammation, synaptic dysfunction, and blood-brain barrier alterations are important components of AD pathogenesis, the AT(N) biomarker matrix is expanding toward an ATX(N) system, where X represents candidate biomarkers for additional pathophysiological mechanisms (*21*). As microglial dysfunction is critical for the development of AD, a biomarker to represent the functional status of microglia is needed. In our present study, plasma sCD22 was elevated in participants at the preclinical AD stage and further increased at the AD dementia stage. Changes in sCD22 were also correlated with core AD pathological hallmarks, baseline cognitive impairment, and longitudinal cognitive decline, suggesting that plasma sCD22 is a potential “X” candidate biomarker to reflect the microglial dysfunctions of AD. This issue is worth investigating in the future.

The strengths of the current study are that the findings were replicated in two independent and geographically dispersed cohorts and that all participants were confirmed with CSF AD biomarkers or Aβ-PET, allowing us to study definitive CN and AD participants at different stages. All these factors improved the reliability of our findings. There are some limitations in our study. First, CD22 is also expressed on mature B cells, and brain CD22 levels were not measured in our study. Whether plasma sCD22 levels can accurately represent the sCD22 levels and functional status of microglia in the brain remains unknown. Second, as this is an observational study, we could not confirm the causal relationship between sCD22 and AD. Third, we could not compare plasma sCD22 with other biomarkers to determine its relative diagnostic value for AD. Furthermore, CN participants in the Chongqing cohort were patients with benign prostatic hyperplasia, stress incontinence, varicose veins of the lower limbs, and rectal polyps and were less frequent in females, which is related to the risk of AD. Participants in the preclinical AD and AD dementia groups of AIBL were older and had a higher frequency of APOE ε4 carriers as compared with the CN participants. These might have a potential impact on plasma sCD22 levels.

In conclusion, our study provides human evidence to support the critical roles of CD22 in AD pathogenesis, suggesting that CD22 may be a potential diagnostic biomarker and therapeutic target for AD. Further studies are warranted to fully understand the roles of CD22 in the pathogenesis of AD and its potential in the diagnosis and treatment of AD.

## MATERIALS AND METHODS

### Study participants

#### Chongqing cohort

All participants in the Chongqing cohort were recruited from the Chongqing Ageing & Dementia Study (CADS). This is an ongoing cohort study initiated in 2010 that aimed to explore the mechanisms of the evolution of aging to AD to identify biomarkers of early diagnosis and interventional strategies for AD. Patients with AD dementia were enrolled from the Neurology Department of Daping Hospital. The clinical assessment and diagnosis of AD dementia were conducted following the protocol used previously (*22*). Briefly, the cognitive and functional statuses of participants with memory and cognitive complaints were assessed using a neuropsychological battery including the MMSE, Montreal Cognitive Assessment, Activities of Daily Living, Auditory Verbal Learning Test, Clock Drawing Test, Trail Making Test, Boston Naming Test, Digit Span Test, Clinical Dementia Rating, Pfeiffer Outpatient Disability Questionnaire, and Hachinski Ischemic Score, and participants were further subjected to PiB-PET to detect the load of brain Aβ deposition. The diagnosis of AD was made according to the recommendations from the National Institute on Aging–Alzheimer’s Association workgroups on diagnostic guidelines for AD (*23*).

Preclinical AD and CN participants were recruited from individuals who underwent lumbar anesthesia for surgical treatment of noninflammatory diseases, including benign prostatic hyperplasia, stress incontinence, varicose veins of the lower limbs, and rectal polyps. CSF samples were collected during lumbar anesthesia before surgery. In the CADS cohort, the cutoff values to define abnormal CSF core AD biomarkers were ≤930.35 pg/ml for Aβ42 (A^+^), >48.56 pg/ml for p-tau 181 (T^+^), and >284.53 pg/ml for t-tau (N^+^). Preclinical AD was defined as normal cognition but abnormal AD core biomarkers, and CN was defined as normal cognition and AD core biomarkers according to the Alzheimer’s continuum category (*24*, *25*).

Participants were excluded if they had (i) concomitant neurological disorders potentially affecting cognitive function; (ii) severe cardiac, pulmonary, hepatic, renal, or neoplastic disorders; (iii) a family history of genetic diseases; and (iv) refused to participate in the study.

Last, a total of 46 patients with AD dementia, 46 patients with preclinical AD, and 66 CN participants from the CADS cohort were included in the present study. Written consent was obtained from all participants or their legal representatives. The study was approved by the Institutional Review Board of Daping Hospital.

#### AIBL participants

The AIBL study is a large-scale longitudinal study of aging, neuroimaging, biomarkers, lifestyle, and clinical and neuropsychological analysis (www.aibl.csiro.au). Participants in the AIBL study were followed up for 90 months with visits from baseline at 18-month intervals to determine the predictive utility of biomarkers, cognitive parameters, and lifestyle factors as indicators of AD and future cognitive decline. Detailed information about the study design, participant recruitment, and clinical assessments was previously described (*26*). AD diagnosis was made according to the recommendations from the National Institute on Aging–Alzheimer’s Association workgroups on diagnostic guidelines for AD (*23*). The AIBL-PACC, consisting of the MMSE, Wechsler Adult Intelligence Scale-Revised Digit Symbol Coding, Wechsler Memory Scale-Revised Logical Memory delayed recall, and the Free and Cued Selective Reminding Test (free recall plus total recall), was used as a measure of cognitive change over time (*27*). EM was chosen as another measure of cognitive change over time (*28*). All participants were subjected to brain Aβ-PET imaging following the protocol described previously (*29*). The AIBL study was approved by the institutional ethics committees of Austin Health, St. Vincent’s Health, Hollywood Private Hospital, and Edith Cowan University. All participants gave written informed consent before entering the study.

A total of 339 AIBL participants were included in the present study. Among them, 138 Aβ-PET^−^ cognitively normal participants were classified as CN controls, 173 Aβ-PET^+^ cognitively normal participants were classified as preclinical AD, and 28 patients with Aβ-PET^+^ AD were classified as AD dementia.

### AD core biomarkers and sCD22 measurements

Fasting CSF samples and blood samples were subjected to centrifugation immediately after being drawn and stored at −80°C until use. Aβ42, Aβ40, t-tau, and p-tau levels in the CSF of the Chongqing cohort were determined using commercially available enzyme-linked immunosorbent assay (ELISA) kits (Innotest, Fujirebio Europe, Ghent, Belgium) in the laboratory of the Neurology Department in Daping Hospital, which joined the Alzheimer’s Association quality control program. Plasma levels of sCD22 were measured using human sCD22 ELISA kits (Antigenix America Inc., Huntington Station, NY) following previously published methods (*13*, *30*, *31*). The linearity of the dilution of the sCD22 ELISA kit was shown in fig. S3, and the linear regression analysis indicated the best-fit line of *y* = 1.1557*x*-89.624 (*R*^2^ = 0.995). The intra-assay precision was ≤11.42% coefficient of variation (table S4), thus demonstrating good reproducibility between sCD22 ELISAs. The spike-and-recovery assessment showed that the recovery rates of sCD22 in plasma at the low spike, median spike, and high spike of the standard sCD22 sample were 91.82, 89.61, and 88.43%, respectively (table S5), indicating that the sample matrices had minimal effect on the ability of the assay to accurately quantify sCD22. ELISA measurements were performed by experienced laboratory technicians who were blinded to the clinical information about the samples.

### Statistical analyses

Baseline characteristics with continuous variables were described as medians/means where appropriate, and categorical data were summarized as absolute frequencies. Differences in the frequencies of sex and APOE ε4 categories were assessed by the chi-square test. Comparative group *P* values were determined by an independent-samples *t* test, Mann-Whitney *U* test, one-way analysis of variance (ANOVA), or ANCOVA with adjustment for age, sex, APOE ε4 genotype, and comorbidities. Correlations of sCD22 with AD biomarkers, brain Aβ-PET SUVR, and baseline cognition were tested by partial correlation analyses adjusted by age, sex, APOE ε4 genotype, and comorbidities, and *r* represents the partial correlation coefficient. Linear mixed models were used to examine the influence of low/high sCD22 levels (split via the median) on cognitive decline over time adjusted for age, sex, and APOE ε4 genotype. Statistical analyses were performed using SPSS (version 20) and GraphPad Prism (version 7.0a). Two-sided *P* < 0.05 was used to define statistical significance.

## Notes

View/request a protocol for this paper from *Bio-protocol*.
