# Supplementary material for: Associations of plasma soluble CD22 levels with brain amyloid burden and cognitive decline in Alzheimer’s disease
Source: Sci Adv. 2022 Apr 1;8(13):eabm5667. doi: 10.1126/sciadv.abm5667 (PMC10938586; doi:10.1126/sciadv.abm5667)
Supplement: Supplementary file 1 — Tables S1 to S5 Figs. S1 to S3 [file sciadv.abm5667_sm.pdf]

Science Advances
AAAS

Supplementary Materials for

Associations of plasma soluble CD22 levels with brain amyloid burden and cognitive decline in Alzheimer's disease

Xian-Le Bu, Pu-Yang Sun, Dong-Yu Fan, Jun Wang, Hao-Lun Sun, Yuan Cheng, Gui-Hua Zeng, Dong-Wan Chen, Hui-Yun Li, Xu Yi, Ying-Ying Shen, Luke A. Miles, Paul Maruff, Ben J. Gu, Christopher J. Fowler, Colin L. Masters*, Yan-Jiang Wang*

*Corresponding author. Email: c.masters@florey.edu.au (C.L.M.); yanjiang_wang@tmmu.edu.cn (Y.-J.W.).

Published 1 April 2022, *Sci. Adv.* **8**, eabm5667 (2022)
DOI: 10.1126/sciadv.abm5667

The PDF file includes:

Tables S1 to S5
Figs. S1 to S3
Legend for data S1

Other Supplementary Material for this manuscript includes the following:

Data S1

Supplementary Materials

Supplementary tables

Table S1. Demographics and clinical data of the participants in the Chongqing cohort.

Variable	CN (n=66)	Preclinical AD (n=46)	AD dementia (n=46)	P values
Age, mean (SD), y	66.3 (7.4)	68.0 (12.7)	66.9 (10.1)	0.674
Female, N (%)	7 (10.6)	14 (26.1) [a]	23 (50.0) [b, c]	<0.001
Education, median (IQR), y	12 (9 - 12)	12 (9 - 12)	10 (6 - 15)	0.526
APOE ε4 carriers, N (%)	12 (18.2)	16 (34.8)	21 (45.7) [b]	0.007
MMSE, median (IQR)	28 (26 - 29)	26 (25 - 29)	16 (12 - 20) [b, c]	<0.001
CSF biomarkers				
Aβ42, mean (SD), pg/ml	1450.1(272.4)	720.4(162.3)	NA	<0.001
Aβ40, mean (SD), pg/ml	11047.2(3152.65)	7738.6(3888.0)	NA	<0.001
T-tau, mean (SD), pg/ml	146.7(50.8)	258.9(130.7) [a]	NA	<0.001
P-tau, mean (SD), pg/ml	36.6(8.0)	48.1(21.7) [a]	NA	<0.001

Results are shown as mean (SD), median (IQR) or number (%).

Abbreviations: AD, Alzheimer's disease; CN, cognitively normal non-AD participants; y, years; MMSE, Mini–Mental State Examination; N, number; APOE, apolipoprotein E; IQR, inter quartile range; SD, standard deviations. CSF, cerebrospinal fluid; NA, non-applicable; Aβ42, amyloid-β 1-42; Aβ40, amyloid-β 1-40; T-tau, total tau; P-tau, phosphorylated tau 181.

[a] $p<0.05$ compared to CN, [b] $p<0.05$ compared to CN, [c] $p<0.05$ compared to preclinical AD.

Table S2. Demographics and clinical data of the participants in the AIBL cohort.

Variable	Baseline				Longitudinal			
	CN (n=138)	Preclinical AD (n=173)	AD dementia (n=28)	P values	CN (n=107)	Preclinical AD (n=147)	AD dementia (n=28)	P values
Age, mean (SD), y	70.6 (5.3)	74.0 (6.4) [a]	79.6 (6.0) [b, c]	<0.001	70.8 (5.7)	74.5 (6.6) [a]	79.6 (6.0) [b, c]	<0.001
Female, N (%)	81 (58.7)	98 (56.6)	16 (57.1)	0.935	63 (58.9)	81 (55.1)	16 (57.1)	0.835
Education, median (IQR), y	12 (10-15)	12 (11-15)	10 (11 - 15)	0.205	12 (10 - 15)	12 (11 - 15)	12 (10 - 15)	0.450
APOE ε4 carriers, no. (%)	26 (18.8)	51 (29.5) [a]	23 (82.1) [b, c]	<0.001	19 (17.8)	41 (27.9) [a]	23 (82.1) [b, c]	<0.001
MMSE, median (IQR)	29 (28-30)	29 (28 - 30)	20 (14 - 25) [b, c]	<0.001	29 (28 - 30)	29 (28 - 30)	20 (14 - 25) [b, c]	<0.001
PACC, median (IQR)	0.009 (-0.518-0.450)	-0.285 (-0.811-0.258) [a]	-1.754 (-2.216- -1.138) [b, c]	<0.001	0.032 (-0.580 -0.451)	-0.285 (-0.811 -0.258)	-1.754 (-2.216 - -1.138) [b, c]	<0.001
EM, median (IQR)	-0.031 (-0.560-0.478)	-0.278 (-0.832-0.239) [a]	-2.167 (-2.882 - -1.389) [b, c]	<0.001	-0.037 (-0.539 -0.480)	-0.278 (-0.869-0.274) [a]	-2.167 (-2.882 - -1.389) [b, c]	<0.001

Results are shown as mean (SD), median (IQR) or number (%).

Abbreviations: AD, Alzheimer's disease; CN, cognitively normal non-AD participants; y, years; APOE, apolipoprotein E; N, number; IQR, inter quartile range; SD, standard deviations; MMSE, Mini–Mental State Examination; PACC, AIBL–Preclinical Alzheimer Cognitive Composite; EM, episodic memory.

[a] $p<0.05$ compared to CN, [b] $p<0.05$ compared to CN, [c] $p<0.05$ compared to preclinical AD.

Table S3. Comparison of plasma sCD22 levels in the Chongqing and AIBL cohorts.

	Comparisons	Unadjusted [a] P values	Adjusted [b] P values
Chongqing cohort	CN vs. Preclinical AD (1385.1±775.3 pg/ml vs. 1748.7±808.3 pg/ml)	0.033	0.034
	CN vs. AD dementia (1385.1±775.3 pg/ml vs. 1766.7±639.7 pg/ml)	0.024	0.057
AIBL cohort	CN vs. Preclinical AD (1069.2±376.2 pg/ml vs. 1257.6±491.1 pg/ml)	<0.001	<0.001
	CN vs. AD dementia (1069.2±376.2 pg/ml vs. 1635.6±343.3 pg/ml)	<0.001	<0.001
	Preclinical AD vs. AD dementia (1257.6±491.1 pg/ml vs. 1635.6±343.3 pg/ml)	<0.001	<0.001

Results are shown as mean ± SD.

Abbreviations: AD, Alzheimer's disease; CN, cognitively normal non-AD participants;

[a] P values were determined by one-way analysis of variance (ANOVA).

[b] P values were determined by analysis of covariance (ANCOVA) with adjustment for age, sex, APOE ε4 genotype and comorbidities.

Table S4. The intra-assay precision of the sCD22 ELISA kits.

Parameters	Sample 1	Sample 2	Sample 3
Mean (pg/ml)	1454.54	832.03	270.47
SD	85.26	95.00	25.72
CV (%)	5.86	11.42	9.51

Abbreviations: SD, standard deviation; CV, coefficient of variation.

Table S5. Results of spike and recovery experiments of the sCD22 ELISA kits.

Sample (N)	Spike level	Expected	Measured	Recovery (%)
Plasma (7)	Low (250 pg/ml)	236.28	216.96	91.82
	Medium (500 pg/ml)	468.27	419.62	89.61
	High (1000 pg/ml)	945.85	836.37	88.43

Supplementary figures

Fig. S1

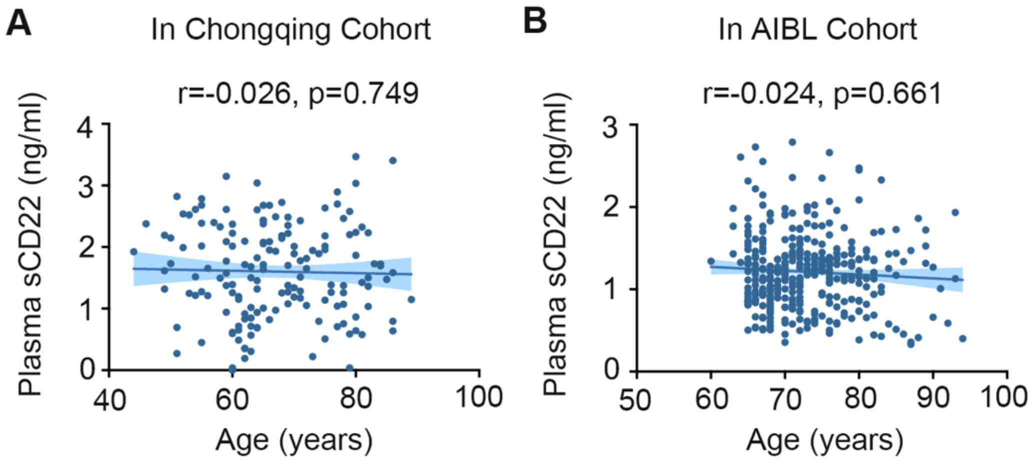

Fig. S1. Correlation of plasma sCD22 levels with age in the Chongqing (A) and AIBL (B) cohorts.

Fig. S2.

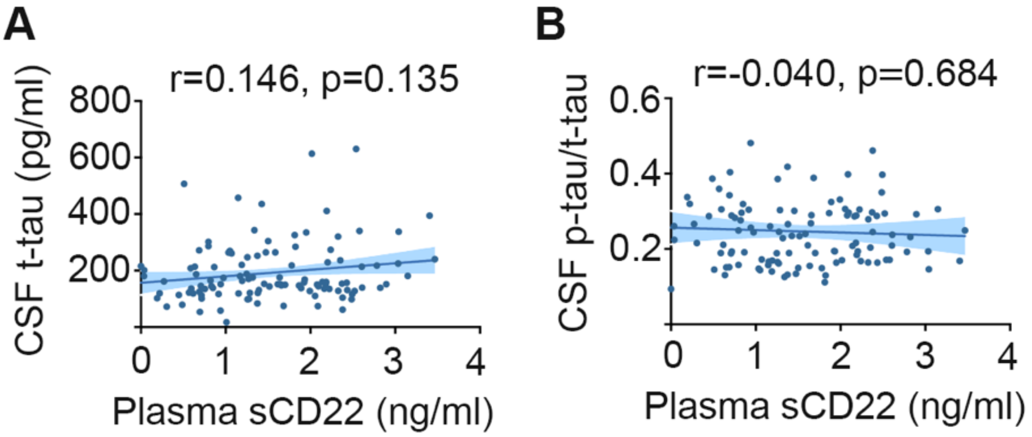

Fig. S2. Correlation of plasma sCD22 levels with CSF t-tau (A) and p-tau/t-tau (B) in the Chongqing cohort. Partial correlations analyses adjusting for age, sex, APOE ε4 genotype and comorbidities. The shaded areas represent the 95% confidence intervals.

Fig. S3.

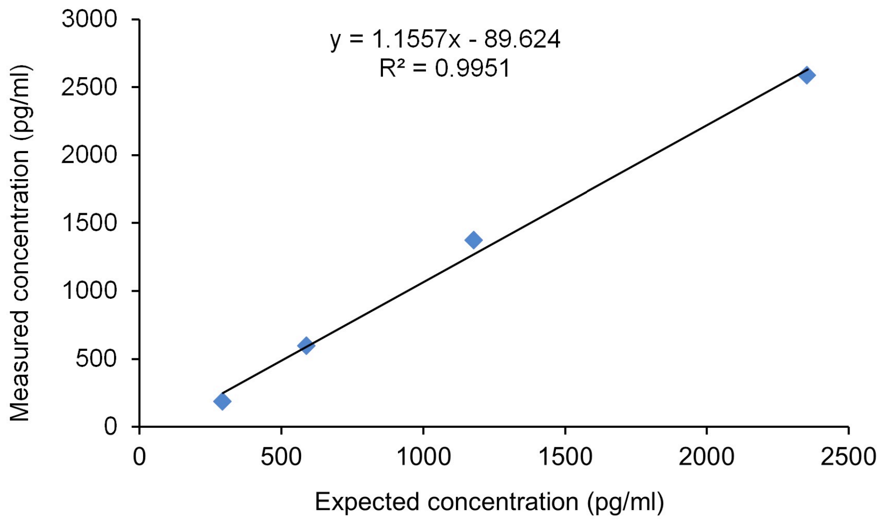

Fig. S3. The linearity of concentration response upon dilution in sCD22 ELISA.

The linear regression analysis indicated the best-fit line of y=1.1557x-89.624 (R^2=0.995).

Data S1. (separate file)

Summary of the plasma sCD22 levels in the Chongqing and AIBL cohorts.
